# Y-90 SIRT: evaluation of TCP variation across dosimetric models

**DOI:** 10.1186/s40658-021-00391-6

**Published:** 2021-06-10

**Authors:** Benjamin J. Van, Yuni K. Dewaraja, Mamadou L. Sangogo, Justin K. Mikell

**Affiliations:** 1 Department of Radiology, University of Michigan, Ann Arbor, MI 48109 USA; 2 Department of Radiation Oncology, University of Michigan, Ann Arbor, 48109 MI USA

**Keywords:** Y-90 Dosimetry, SIRT, Partition Model, Radioembolization

## Abstract

**Introduction:**

Much progress has been made in implementing selective internal radiation therapy (SIRT) as a viable treatment option for hepatic malignancies. However, there is still much need for improved options for calculating the amount of activity to be administered. To make advances towards this goal, this study examines the relationship between predicted biological outcomes of liver tumors via tumor control probabilities (TCP) and parenchyma via normal tissue complication probabilities (NTCP) given variations in absorbed dose prescription methodologies.

**Methods:**

Thirty-nine glass microsphere treatments in 35 patients with hepatocellular carcinoma or metastatic liver disease were analyzed using ^99m^Tc-MAA SPECT/CT and ^90^Y PET/CT scans. Predicted biological outcomes corresponding to the single compartment (standard) model and multi-compartment (partition) dosimetry model were compared using our previously derived TCP dose-response curves over a range of 80–150 Gy prescribed absorbed dose to the perfused volume, recommended in the package insert for glass microspheres. Retrospective planning dosimetry was performed on the MAA SPECT/CT; changes from the planned infused activity due to selection of absorbed dose level and dosimetry model (standard or partition) were used to scale absorbed doses reported from ^90^Y PET/CT including liver parenchyma and lesions (*N* = 120) > 2 ml. A parameterized charting system was developed across all potential prescription options to enable a clear relationship between standard prescription vs. the partition model-based prescription. Using a previously proposed NTCP model, the change in prescribed dose from a standard model prescription of 120 Gy to the perfused volume to a 15% NTCP prescription to the normal liver was explored.

**Results:**

Average TCP predictions for the partition model compared with the standard model varied from a 13% decrease to a 32% increase when the prescribed dose was varied across the range of 80–150 Gy. In the parametrized chart comparing absorbed dose prescription ranges across the standard model and partition models, a line of equivalent absorbed dose to a tumor was identified. TCP predictions on a per lesion basis varied between a 26% decrease and a 81% increase for the most commonly chosen prescription options when comparing the partition model with the standard model. NTCP model was only applicable to a subset of patients because of the small volume fraction of the liver that was targeted in most cases.

**Conclusion:**

Our retrospective analysis of patient imaging data shows that the choice of prescribed dose and which model to prescribe potentially contribute to a wide variation in average tumor efficacy. Biological response data should be included as one factor when looking to improve patient care in the clinic. The use of parameterized charting, such as presented here, will help direct physicians when transitioning to newer prescription methods.

**Supplementary Information:**

The online version contains supplementary material available at 10.1186/s40658-021-00391-6.

## Introduction

Selective internal radiation therapy (SIRT) is an increasingly widespread treatment for liver cancers across the globe [1]. Unresectable hepatocellular carcinoma (HCC), metastatic colorectal cancer (CRC), and neuroendocrine tumor metastases (NET) are commonly treated with SIRT using Yttrium-90 (^90^Y) labeled microspheres with resin (SIR-Spheres®) or ^90^Y within a glass matrix (TheraSphere®). The introduction of glass microsphere treatments in the late 1980s and early 1990s led to clinical trials and its approval for use in the USA treating HCC and other metastatic liver cancers under FDA Humanitarian Device Exemption [2]. Despite the vast progress already made in implementing SIRT as a viable treatment option, a recent multinational survey of SIRT physicians shows that better estimates of the microsphere distribution in the liver and improved options for calculating the amount of activity to be injected were among the most requested improvements for their practice [3].

Current methods for prescribing activity to tumor sites within the liver vary across practices and devices. Recommendations for glass microspheres given by the TheraSphere Global Dosimetry Steering Committee suggest either a single-compartmental (standard model) or multi-compartmental (partition model) dosimetric model for calculating dose to tumor and normal liver depending on treatment intent and patient selection [1]. With the tumors and surrounding normal liver of the injected lobe grouped into a single volume of interest, the standard model makes the absorbed dose calculations straightforward, but these calculations may not always be indicative of the absorbed dose deposited in each lesion or absorbed dose to normal liver. The partition model (PM) [4] keeps the assumption of uniformly distributed activity over a volume as in the standard model, but expands the volume definitions from a single target volume into two separate quantities: the tumoral liver and the normal liver that allow for improved estimation of dose delivered to cancerous regions versus normal functioning liver in the segments supplied by the infused artery. In addition to the compartmentalized dosimetric evaluation, more rigorous options include voxel-based assessments which may be of use in the future [5]. One method of obtaining pre-treatment voxelized information is through the use of ^99m^Tc-MAA SPECT scans. The ^99m^Tc-MAA is primarily used in the standard model for estimating extrahepatic shunting. For the partition model, the ^99m^Tc-MAA SPECT/CT is also useful for measuring relative uptake of lesions to liver parenchyma. However, the ^99m^Tc-MAA SPECT/CT is not yet widely implemented as a predictive measure at the voxel level, as the quality of the ^99m^Tc-MAA distribution representation of the ^90^Y microsphere distributions has been debated with some studies suggesting adequate [6] and others finding insufficient [7] correlation.

For the dose prescription process, which like many medical choices relies partly on recordable features like tumor stage, liver function, presence of cirrhosis, treatment intent, and partly on physician gestalt, relating a traditional methodology to a new procedure may be tedious. The aim of our work is to provide a comparison of the predicted biological dose-response for ^90^Y glass microsphere lobar liver treatments between the standard model and the partition model. As the range of absorbed doses available to physicians is quite broad when prescribing via the current TheraSphere guidelines (80–150 Gy) [8], a detailed investigation into how the TCP predictions change over this range was conducted. A parameterized charting system was developed across all potential prescription options to enable a clear relationship between traditional package insert prescription vs. the partition model method. This data will help guide clinical decision-making among practices looking to transition from the standard model to the partition model when determining prescribed activity.

## Methods and materials

### Patient characteristics

Patients presenting with HCC, CRC, NET, melanoma, and other metastatic lesions enrolled in ^90^Y treatment at Michigan Medicine from 2016 to 2019 were examined for this study. A total of 35 patients were treated covering 120 lesions with ^90^Y glass microsphere intraarterial injections. These injections ranged from 0.5 to 12.6 GBq per treatment. Patients receiving treatment to both the right and left lobes were considered independent cases, for a total of 39 treatments. Patients included in this study had a minimum of 1 and a maximum of 9 tumors each that ranged in size from 2 to 871 ml. Subjects with HCC, all cirrhotic, comprised 38% of cases and 36% of all lesions. Among metastatic diseases, colorectal, adrenal, and cholangiocarcinoma lesions were the most commonly included with 4 patients each. Melanomas and neuroendocrine liver metastasis were treated in 3 patients, and pancreatic and appendiceal metastasis had one patient in this study apiece. All patients were Child-Pugh class A at baseline prior to treatment.

### Imaging

All patients underwent pretreatment ^99m^Tc-MAA planar and SPECT/CT imaging on Siemens Symbia systems (Intevo or T series) for clinical purposes to estimate lung shunt and extra-hepatic deposition. The acquisition parameters were 15% photopeak and an adjacent 15% scatter window; 128 × 128 matrix, 4.8 mm pixels, 60 views/head; non-circular orbit; and 10–20 min acquisition time. The images were reconstructed using 8 iterations, 4 subsets of Siemens 3D-OS-EM software (Flash3D, Siemens Medical Solutions, Malvern, PA) including CT-attenuation correction, triple-energy window scatter correction, resolution recovery, and a 8.4-mm Gaussian post-filter. The voxel size was 4.8mm^3^. The CT was performed in low dose mode (130 kVp; 80 mAs) during free-breathing.

Patients in the study signed an informed consent document to participate in the research ^90^Y imaging. Post-treatment ^90^Y imaging within 4 h after microsphere injection was performed via a Siemens Biograph mCT PET/CT with time-of-flight (TOF resolution 530 ps). Patient PET data were reconstructed with Siemens 3D-OSEM software using the following parameters that were chosen based on a previous [9] ^90^Y phantom evaluation: TOF, 1 iteration 21 subsets, attenuation correction, scatter correction, randoms correction, resolution recovery, and a 5-mm Gaussian post-filter. The PET matrix size was 200 × 200 with a pixel size of 4.07 × 4.07 mm^2^, and slice thickness of 3 mm. The CT was performed in low dose mode (120 kVp; 80 mAs) during free-breathing.

The perfused liver volume was defined via pre-treatment angiography and approved by an interventional radiologist. Each perfused liver volume is composed of the liver segments or lobe being supplied by the artery to be infused [10]. Tumor volumes > 2 mL were segmented manually on baseline diagnostic CT or MR by an experienced radiologist specializing in hepatic malignancies. The diagnostic CT or MR was then rigidly registered to the co-registered ^99m^Tc-MAA SPECT/CT and ^90^Y SPECT/CT and contours were transformed within MIM version 6.9 (MIM Software Inc., Cleveland, OH). Fine manual adjustments were made as needed to account for mis-registration. The liver was segmented directly on the CT of SPECT/CT and PET/CT using deep learning or atlas-based semi-automatic tools within MIM. The healthy liver was defined as the liver minus the contoured lesions.

**Fig. 1:**
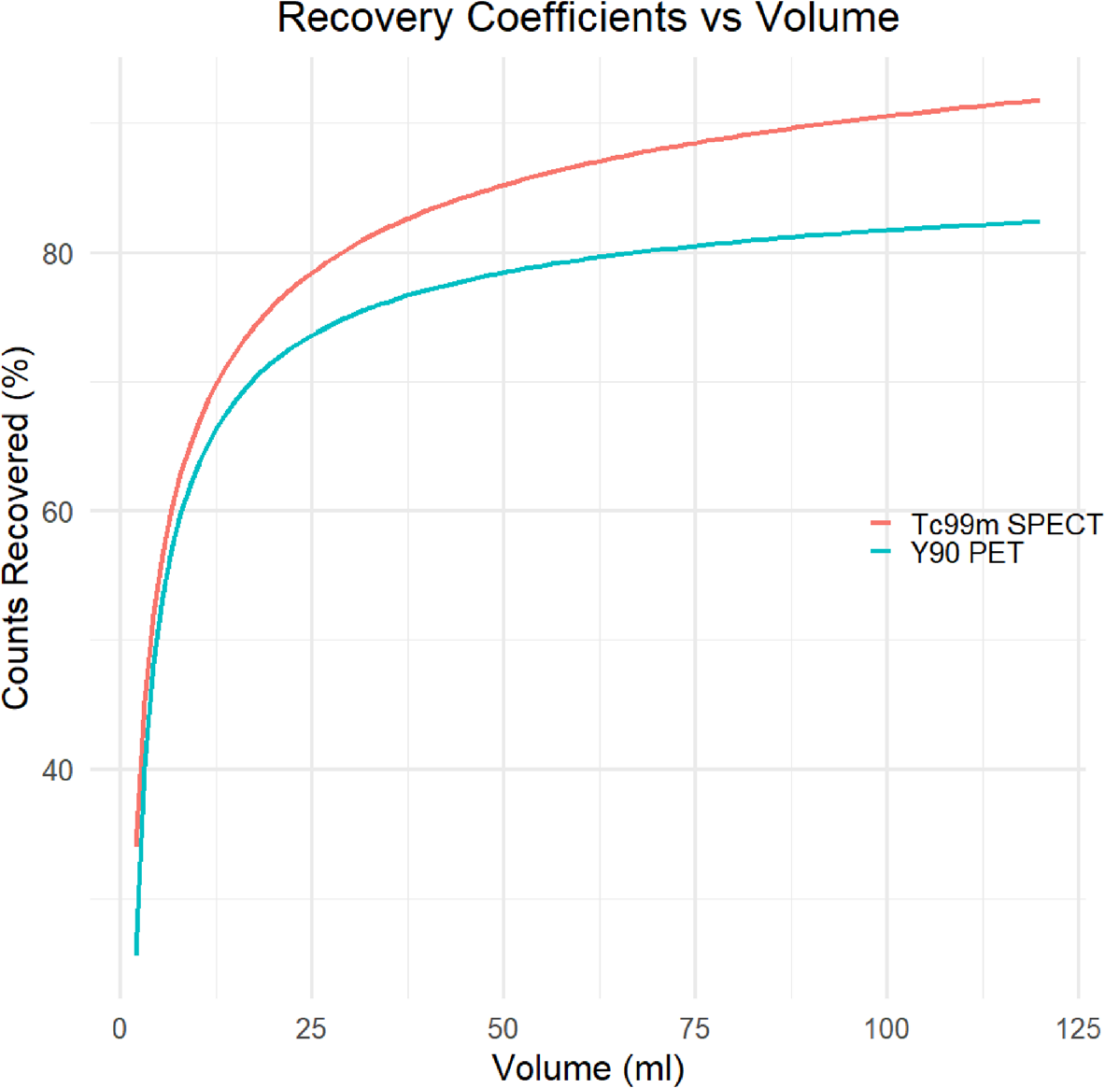
Recovery coefficient curves for ^90^Y PET and ^99m^Tc-MAA SPECT. Phantom acquisition and reconstruction parameters were as described for the patient studies

In order to correct for partial volume effects that are a consequence of finite spatial resolution, volume-dependent recovery coefficients (RCs) were applied to mean counts in lesions and non-tumoral liver for both SPECT and PET. The RCs for ^90^Y were measured as part of a previous study [9] and were repeated for Tc-99 m using the same phantom setup (6 spheres of volume 2 – 113 mL with a sphere-to-background ratio of 9:1). The fitted RC vs. volume curves from these measurements are shown in Fig. 1.

### Dosimetry via the single compartment model

The current standard for dosimetry in ^90^Y SIRT is based on the Medical Internal Radiation Dosimetry (MIRD) formalism, which provides an estimate of the average energy deposition per GBq (${\overline{E}}_D$) by utilizing an average beta energy absorption per nuclear transition (${\overline{E}}_{\beta }$) as a substitute for a full transport simulation such as Monte Carlo [11]. This allows for a quick calculation of absorbed dose to the volumes of interest and is appropriate for non-penetrating relatively low energy beta emissions. With an average beta energy of 933 keV per disintegration emitted in the decay of ^90^Y, the majority of the dose is deposited locally around the microspheres. This high percentage of locally deposited energy keeps the non-penetrating MIRD model accurate and is used for all absorbed dose calculations in this study. Under the assumption that the microspheres are permanently lodged inside the liver with no biological clearance and a physical half-life (*T*_1*/*2_) of 2.66 days the total energy deposited in a volume of interest (VOI) can be calculated and scaled based on injected activity.

When all of these factors are accounted for the average energy deposited by ^90^Y is 49.6 ≈50 J/GBq. Assuming non-penetrating radiation and uniform activity for any VOI (e.g., tumor, non-tumoral liver, or perfused lobe), the absorbed dose (*D*_*VOI*_) to the VOI per unit activity injected (A) to that volume of mass (*M*_*VOI*_) containing activity (*A*_*VOI*_) is given by [12]:

1 $$ \frac{D_{VOI}}{A}\left(\frac{Gy}{GBq}\right)=50\ \left(\frac{J}{GBq}\right)\cdotp \frac{\frac{A_{VOI}}{A\kern0.5em }}{M_{VOI}\ (kg)\ } $$

All delivered tumor and normal liver absorbed doses were calculated according to equation 1 using the defined contours, an assumption of density = 1.03 g/cc, and the direct ^90^Y PET/CT image-based activities. Under the assumption that all injected activity is in the infused liver lobe and lung, the injected activity, A, required to deliver a dose D to the lobe can be expressed as:

2 $$ {A}_{Lobe}=\frac{D_{Lobe}(Gy)\ast {M}_{Lobe}(kg)}{50\ \left(J/ GBq\right)} $$

### Multi-compartmental (partition) model

As with the standard model, the partition model uses the MIRD model’s average energy deposition of 50 J/GBq as well as the injected activity scaled by the lung shunt fraction for dose calculations. Instead of a singular lobar or segmental mass, the partition model requires the mass of the normal liver (*M*_*NL*_) and the cumulative mass of all defined lesions (*M*_*T*_). The tumor to normal liver uptake ratio (TNR) can be measured by the tumor uptake of MAA relative to the normal liver parenchyma. Planning or predictive dosimetry uses this ratio to distribute the activity between the mass compartments. In this work, TNR was determined via ^99m^Tc-MAA pretreatment SPECT scans for the radiologist defined tumor and non-tumoral liver. Selecting an absorbed dose for normal liver D given the masses of the normal liver and tumor compartments and lung shunt fraction, an injected activity A, can be calculated according to equation 3. For an injected activity A, these can be used to calculate the absorbed dose per unit activity for either the normal liver, as seen in equation 4, or to the tumor, equation 5 [12].

3 $$ A=\frac{D_{NL}(Gy)\ast \left({M}_{NL}(kg)+ TNR\ast {M}_T(kg)\right)\ }{50\ \left(J/ GBq\right)\ast \left(1- LSF\right)} $$

4 $$ \kern0.75em \frac{D_{NL}}{A}\left( Gy\  per\  GBq\right)=50\ \left(J/ GBq\right)\cdotp \frac{\left(A\cdotp \left(1- LSF\right)\right)/A}{M_{NL}\ (kg)+ TNR\cdotp {M}_T\ (kg)} $$

5 $$ \kern0.5em \frac{D_T}{A}\left( Gy\  per\  GBq\right)=50\left(J/ GBq\right)\cdot \frac{\frac{A\cdot \left(1- LSF\right)}{A}\cdot TNR}{M_{NL}(kg)+ TNR\cdot {M}_T\ (kg)} $$

### Absorbed dose parameterization for TCP comparison

**Fig. 2:**
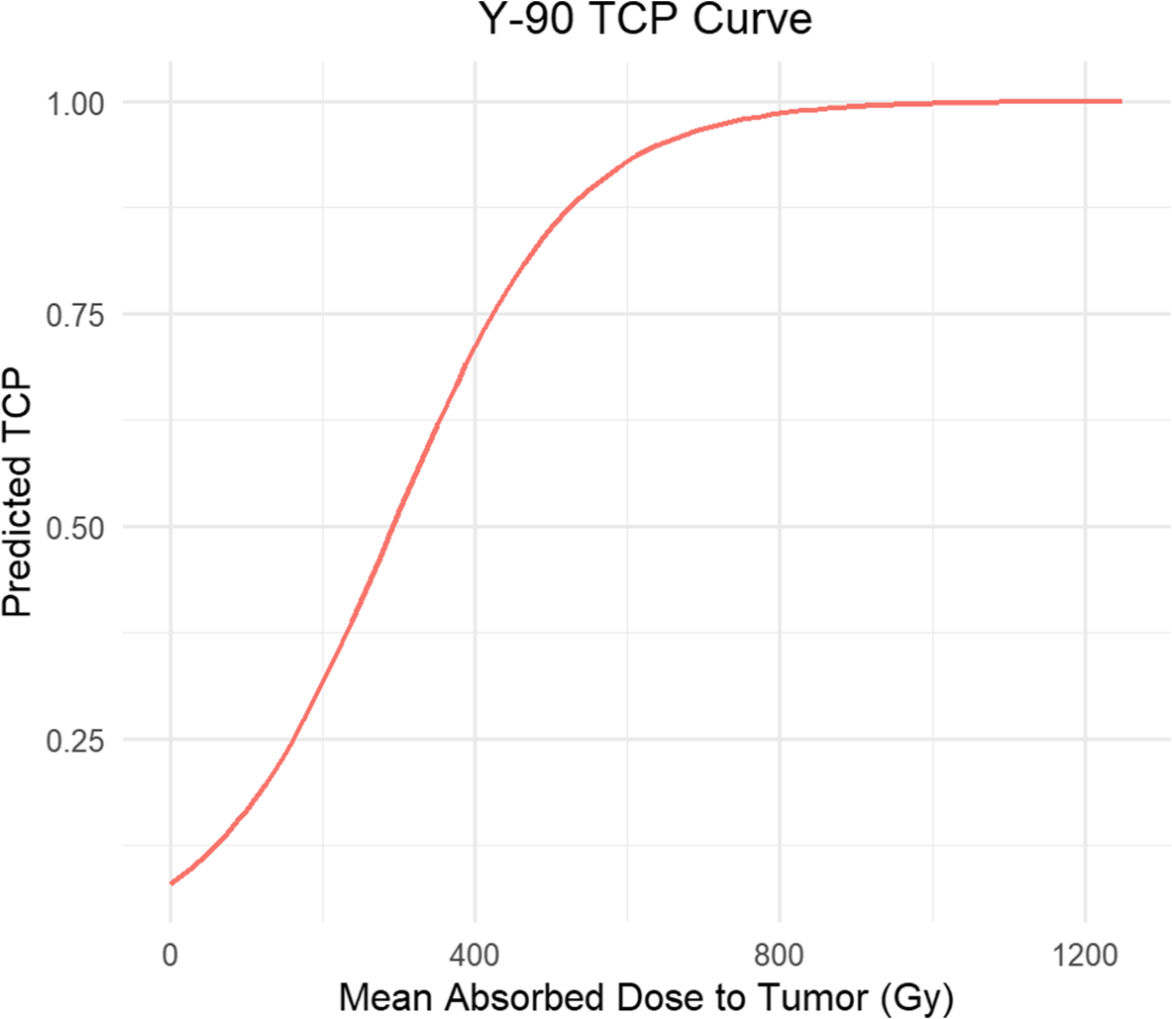
Fitted tumor control probability model for mean tumor dose from prior study [9]

In order to observe changes in TCP corresponding to prescribing ^90^Y using the standard model versus the partition model, a parameterized chart of absorbed dose ranges was created for the retrospective analysis of 120 lesions over 35 patients. The TCP curve (Fig. 2) used in this work was from our prior report [9] where a logit model was used to fit mean tumor absorbed dose-response data in patients who underwent ^90^Y PET/CT imaging following SIRT at our institution.

As per the package insert [8], the standard model prescription absorbed dose ranges were allowed to vary between 80 and 150 Gy to the infused liver volume depending on tumor staging, cirrhosis status, and estimated percentage of the microspheres that will end up shunted to the lung (LSF), as well as the estimated nominal residual activity in vial and tubing. Partition model prescription ranges were chosen by consulting the TheraSphere Global Dosimetry Steering Committee guidelines of 75 Gy to the normal liver for lobar treatments [1] as well as NTCP curve data showing close to 100% normal tissue complications being reached at 150 Gy to the normal liver [5]. These numbers served as a rough guideline for choosing the 40–150 Gy absorbed dose range of prescriptions presented in this paper.

For each of the prescribed dose values to the perfused liver volume (for SM) and normal liver (for PM), the hypothetical injected activity A was calculated using equations 2 and 3. The corresponding absorbed dose to individual tumors (not the entire compartment) was calculated by scaling the previously calculated absorbed dose per injected activity from the post-therapy 90Y PET/CT by this hypothetical injected activity using equation 1. Then, using our previously generated curve of Fig. 2, the TCP corresponding to each tumor absorbed dose value was calculated for the SM and PM and the change in TCP between the two prescription models was determined. This process was repeated for every combination of SM and PM prescriptions to generate a parameterization chart.

### NTCP Comparison

The predicted normal tissue outcomes were calculated via the new radiobiologic model, equation 8, put forth by Walrand et al. [13, 14]. For the current study, the whole liver absorbed dose that gives NTCP = 0.15 (WLTD15) was calculated using equations 6, 7, and 8 and was compared with the whole liver dose associated with a standard model 120 Gy absorbed dose prescription to the infused volume. This model uses external beam radiotherapy treatment (EBRT) clinical data with an endpoint of radiation-induced liver disease. The WLTD was calculated assuming a NTCP (p) for microsphere treatments according to the volume fraction of the liver that is targeted (*Vf*), the killed lobule fraction (*Kf*), and the specific activity of the microspheres at the time of injection (*msA*):

6 $$ Kf(p)=0.4\ast \sqrt[8.29]{\frac{p}{1-p}} $$

7 $$ \kern1.75em F(msA)=\left(1-{e}^{-\sqrt[3]{\frac{msA}{0.0471 kBq}}}\right) $$

8 $$ WLTD\left(p, Vf,\mathrm{m} sA\right)=47.1\  Gy\ast \frac{\left(1+0.457\ p\right)\ast F(msA)}{{\left( Vf- Kf(p)\right)}^{0.869\ast F(msA)}}\ast Vf $$

where F is a dimensionless scale factor that is a function of msA and is related to the average inter-microsphere distance [14].

The NTCP model is based on previous work from external beam therapy showing normal tissue complications to be near zero as long as the portion of treated liver was under 25% to 40% of the total liver volume [15]. Patients with segmentectomies or smaller lobular targets were therefore not included in the NTCP prediction data, as normal tissue complications are not expected to impact patient care in these cases due to the nature of the liver as a parallel organ and small treatment area. In this study, only 29 of the 39 treatments had targeted liver volume fractions large enough (> 40% of the total liver) for the calculation of NTCP.

## Results

**Fig. 3:**
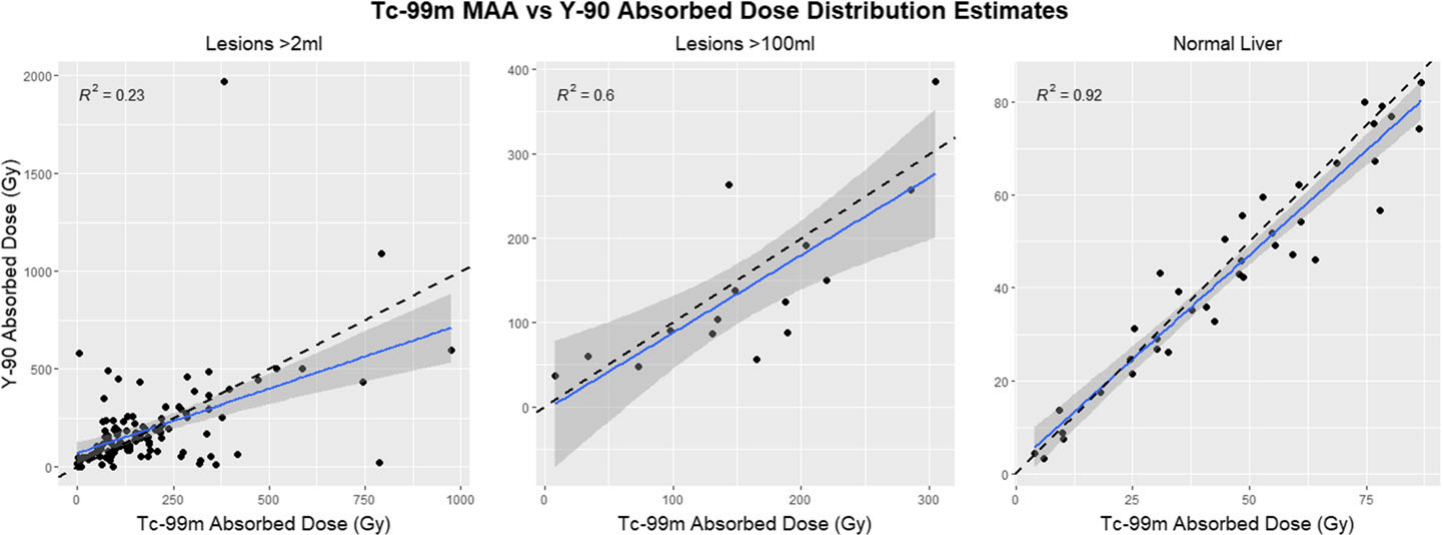
Comparison between absorbed dose estimates for ^99m^Tc-MAA SPECT and ^90^Y PET. The dashed line denotes the identity line while the solid line shows the current linear fit

Plots of the ^99m^Tc-MAA SPECT/CT-based absorbed dose predictions vs. the delivered absorbed doses based on ^90^Y PET/CT, Fig. 3, show varying levels of correlation depending on the size of the investigated volume. With smaller treated lesion volumes included the correlation was poor. The subset of lesions with a volume *>* 2 ml recorded an R^2^ of 0.23 via a linear fit. For lesions *>* 100 ml, the R^2^ was 0.60 and when investigating only the normal liver volumes, ranging from 837 to 5049 ml, data showed a good correlation with an R^2^ value of 0.92 between ^99m^Tc-MAA SPECT and PET dose predictions.

**Fig. 4:**
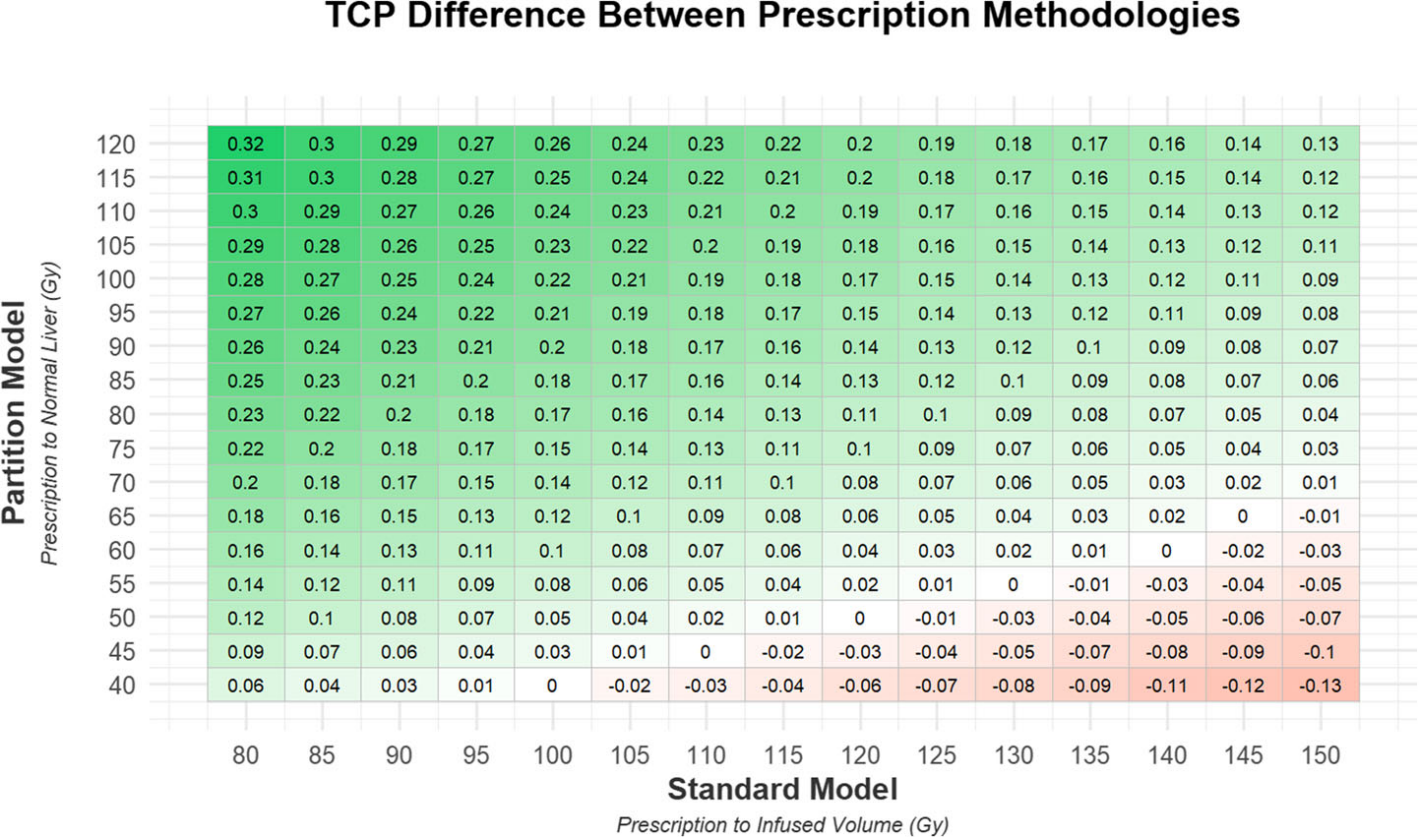
Average change in tumor control probability estimate when moving from the standard model, with the absorbed dose in Gy prescribed to the treatment perfused volume, to the partition model, with the absorbed dose in Gy prescribed to the normal liver

Figure 4 shows the parameterization chart presented as a heat map of TCP with the absorbed dose prescriptions to the infused volume using the standard model on the x-axis and absorbed dose prescriptions to the normal liver using the partition model on the y-axis. This figure is generated using ^99m^Tc-MAA SPECT/CT and appropriately scaled ^90^Y PET/CT data only to demonstrate hypothetical treatment planning and subsequent dose verification results. The average change (across all 120 evaluated lesions) in predicted TCP if the partition model was used instead of the standard model is displayed in each box. At either end of the spectrum, the difference between prescriptions corresponds to a 13% decrease in TCP when prescribing 40 Gy to the normal liver via the partition model instead of 150 Gy to the infused volume via the standard model and to a 32% increase in TCP when prescribing 120 Gy to the normal liver via the partition model instead of 80 Gy to the infused volume via the standard model. All TCP changes can be seen in Fig. 4.

For the most common dose delivered to patients in this study, 120 Gy per the standard model, a prescription of 47 Gy to the normal liver was found to have an equivalent tumor control probability for the partition model. The TheraSphere Global Dosimetry Steering Committee’s suggestion of 75 Gy to the normal liver for lobar treatment showed an increase in TCP of approximately 10% over the standard model 120 Gy prescription.

A line of equivalent TCP, and therefore equivalent prescribed activity and absorbed dose, is evident in the relational heatmap presented in Fig. 4. In our study, prescribing 100 Gy via the standard model gives the same TCP outcome on average as prescribing 40 Gy to the normal liver in the partition model. This pattern holds for 110 Gy SM:45 Gy PM, 120 Gy SM:50 Gy PM, 130 Gy SM:55 Gy PM, and 140 Gy SM:60 Gy PM.

**Fig. 5:**
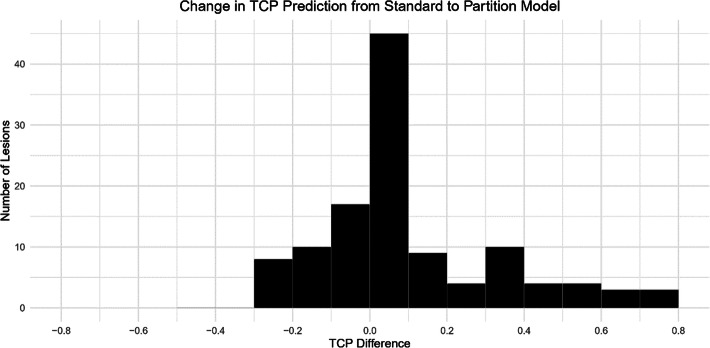
Histogram of change in tumor control probability estimate for individual tumor when prescribing 75 Gy to the normal liver using the partition model vs 120 Gy to a lobe via the standard model

While the average change in TCP was small for many of the prescription variations, there were large shifts in individual tumor control probabilities for certain tumor subsets. Figure 5 shows individual tumor TCP distribution histograms corresponding to 120 Gy absorbed dose prescription to the infused volume and a 75-Gy absorbed dose prescription to the normal liver with per lesion TCP’s varying from -26% to 81% with the standard deviation for this distribution being 23.5%. Further lesion level distributions are available in the supplementary files.

Similarly, large shifts in absorbed dose to the liver and tumors were found on a patient by patient basis when prescribing to 15% NTCP instead of a standard model prescription of 120 Gy. Liver absorbed dose values ranged from a 9% decrease to a 286% increase over the patient population in this study. On average, this turned out to be a 58% increase in dose to the entire liver over the population as a whole.

## Discussion

The predicted outcome probabilities such as the TCP or NTCP values presented in this study are not accurate predictors of patient-specific clinical outcome, but rather an average estimate of dose-response that can be used in conjunction with patient history, disease stage, and physician experience, to guide clinical decision-making. In addition, while the partition model potentially shows improvement in dose estimation and allows for prescription limits on normal tissue in the target volume, it is still a rough estimate, particularly when dealing with multiple lesions that may have different vascularities [16].

One strength of our study is that changes in prescribed activity based on planning dosimetry model and prescribed absorbed dose level on ^99m^Tc-MAA SPECT/CT were applied to individual tumor absorbed doses reported on the patient’s post therapy ^90^Y PET/CT. This allowed us to evaluate the change in TCP for individual lesions and provide population averages. This is important because the mean absorbed dose to normal liver from ^99m^Tc-MAA SPECT/CT can predict ^90^Y PET/CT mean absorbed dose to normal liver well, but predicting delivered mean absorbed dose to tumors is difficult, especially for smaller lesions which are common in lobar directed microsphere treatments. In this study, we explicitly included the discordance between plan and delivered distributions when evaluating change in TCP. A normal liver absorbed dose of 75 Gy via partition model is considered safe for Child-Pugh A cirrhotic patients [2]. Our parameterization suggests that transitioning to planning for normal liver absorbed dose of 75 Gy with partition model from any currently recommended absorbed dose via standard model will increase the TCP on average from at least 3% to 22%.

Similar conclusions have been shown in another retrospective study with Y-90 microspheres. A 2018 study provided a comparison of the BSA dose calculation method to voxel-level dosimetry in resin microspheres and showed that the BSA gave suboptimal activity. Using an optimal activity, the dose to the tumor could be increased for most patients while still staying within recommended thresholds for non-tumoral liver and lung doses [17].

Results from the DOSISPHERE-01 phase 2 clinical trial have also demonstrated the use of personalized dosimetry based on ^99m^Tc-MAA SPECT/CT for treatment planning [18]. The study required at least a 7-cm lesion for calculation of the prescribed dose which corresponds to ~ 180 ml tumor volume. The real-world implementation demonstrated in the DOSISPHERE trial shows the viability of personalized dose prescription models. Further evaluation is still warranted for smaller lesions where lesion segmentation, partial volume effects, and motion errors may complicate planning as evident from the poor correlation we observed between ^99m^Tc-MAA SPECT/CT and ^90^Y PET/CT imaging-based estimates of absorbed dose (Fig. 3).

While large increases in average patient TCP appear possible in Fig. 4 by boosting the dose delivered to the tumor, the tradeoff is always increased posttreatment complications and toxicity. NTCP curves for glass microspheres [5] show that ~ 75 Gy to the normal liver correlates to a 15% chance of complications and raising that prescription to 105 Gy will, on average, increase the complication rate to around 50%. For our standard patients, typically prescribed 120 Gy using the standard model, a 10% increase in average TCP is expected if prescribed 75 Gy using the partition model.

When using Walrand’s NTCP model on our study population, we saw a significant increase of 58% average change in prescribed dose from a standard model prescription of 120 Gy to the infused volume to a 15% NTCP prescription to the normal liver. On a patient-by-patient basis, limiting the prescription on a NTCP basis altered the prescribed dose between − 9% and 286%. Only 2 patients saw a decrease in prescriptions as a result of using the 15% NTCP cutoff.

**Fig. 6:**
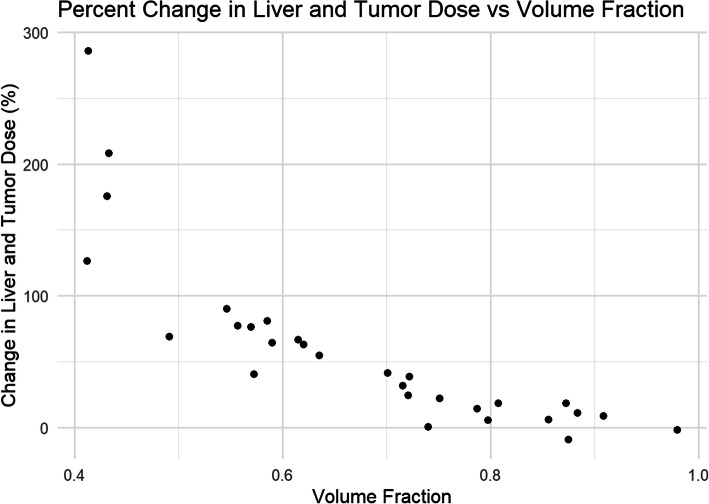
Change in predicted tumor dose and normal liver dose when prescribing to 15% NTCP, instead of a 120 Gy absorbed dose to the infused volume via the standard model, is charted against the liver targeted volume fraction

Figure 6 shows the relationship between the targeted liver volume fraction for the 29 patients that had targeted liver volumes large enough for evaluation using the NTCP model. The general trend is for increased absorbed doses when using the 15% NTCP operating point with smaller targeted liver volume fractions. Over the patients that were evaluable for NTCP, there was room to increase the prescribed dose in all but two cases which had large volume fractions.

The NTCP model has not been validated, and different clinically acceptable complication rates (e.g., 5% vs 15%) would be determined on a case-by-case basis when used for planning. Given the lack of validation, we consider it an example of how models may impact future radioembolization planning. Additional investigation and validation of ^90^Y microsphere liver NTCP models are needed before clinical use. As the community moves towards more personalized treatment planning, evaluation of the risk to normal tissue is a viable metric to include as one of many factors available to the physician when making treatment decisions.

With the analysis of patient toxicity and outcomes in this study, further work may incorporate deciding how to balance dose efficacy and toxicity. One option moving forward is to use the utility approach [19]. This model allows for a quantitative forecast of the optimal prescribed dose by applying a marginal rate of toxicity factor dictated by the physician’s level of comfort when assessing risk to any given patient. This toxicity factor can then be combined with TCP and NTCP data to balance treatment effectiveness with normal tissue complications on a case-by-case basis.

An important additional factor affecting toxicity and functional hepatic reserve that could be included is the baseline liver function. Patients with less liver dysfunction (no cirrhosis or Child-Pugh A) will likely have a different NTCP curve allowing more absorbed dose than those with worse liver function. Such differences were not included in the NTCP but are important and could be included in future utility estimates.

With high variability in predicted vs. delivered absorbed doses for small tumors, a certain amount of caution should be used if ^99m^Tc-MAA-based predictions will be used for clinical pretreatment decision-making on a patient specific basis. We found that smaller tumors did not show a good correlation between the ^99m^Tc-MAA SPECT and posttreatment ^90^Y PET scans. However, we did find an improved correlation between pre- and post-emission imaging for larger lesions (Fig. 3) and a good to excellent correlation for non-tumoral liver. This is similar to prior studies suggesting or demonstrating ^99m^Tc-MAA partition model planning tumor dosimetry being more reliable for larger lesions.

Similar to our work, Thomas et al. reported large errors in predicting delivered absorbed dose to multiple tumors when using the partition model [20]. This does not significantly affect the overall conclusions of this study as the TCP data presented is a bulk average of all lesions and not indicative of individual lesion response. In order to verify this claim, a retrospective analysis repeating the work done in this paper, but substituting posttreatment ^90^Y PET scans in place of the pretreatment ^99m^Tc-MAA SPECT, was completed. This analysis found that variation between the SPECT and PET data did not cause large changes in average TCP calculations, with less than a 1% deviation in any of the data shown in Fig. 4. Our data suggests that the use of the ^99m^Tc-MAA SPECT on an individual basis for prescription to the normal liver volumes via partition model calculations is reasonable, as larger volumes correlate well with posttreatment ^90^Y PET data.

Segmentation of tumor and nontumoral regions plays an important role in partition model dosimetry. Diffuse, infiltrative, or widespread tumors and the corresponding nontumoral regions may be difficult or impractical to segment. The use of additional functional imaging such as ^99m^Tc-sulfur colloid imaging may help when boundaries are not clear on anatomical imaging [21]. Only clearly defined tumors were evaluated during this study.

## Conclusion

The use of biological dose-response factors can be a valuable asset in guiding clinical decision-making in selective internal radiation therapy. We have shown that the choice of the prescribed dose and which model to prescribe by may contribute to a wide variation in average tumor efficacy for patients. These relationships between the standard model and partition model should be considered alongside the other advantages and disadvantages of each model when making changes to liver radiotherapy care for patients. To that end, the derived parameterized prescription charts will allow for the ability to concisely weigh the impact that a change in prescription might have on a patient population.

## Supplementary Information

**Additional file 1: Figure S1.** The standard deviation is shown for each average change in TCP calculated presented in Fig. 4. **Figure S2.** The largest increase in TCP for any lesion when changing from a standard model prescription to a partition model prescription. **Figure S3.** The largest decrease in TCP for any lesion when changing from a standard model prescription to a partition model prescription.

## Data Availability

Anonymized ^90^Y PET/CT DICOM data including segmented lesions for select patients are available at the University of Michigan Library Deep Blue repository: 10.7302/v07v-z854 and 10.7302/pf4m-vn04.
