# Supplementary material for: Y-90 SIRT: evaluation of TCP variation across dosimetric models
Source: EJNMMI Phys. 2021 Jun 10;8:45. doi: 10.1186/s40658-021-00391-6 (PMC8192668; doi:10.1186/s40658-021-00391-6)
Supplement: Supplementary file 1 — Additional file 1: Figure S1. The standard deviation is shown for each average change in TCP calculated presented in Fig. 4. Figure S2. The largest increase in TCP for any lesion when changing from a standard model prescription to a partition model prescription. Figure S3. The largest decrease in TCP for any lesion when changing from a standard model prescription to a partition model prescription. [file 40658_2021_391_MOESM1_ESM.docx]

**Supplemental Figures**


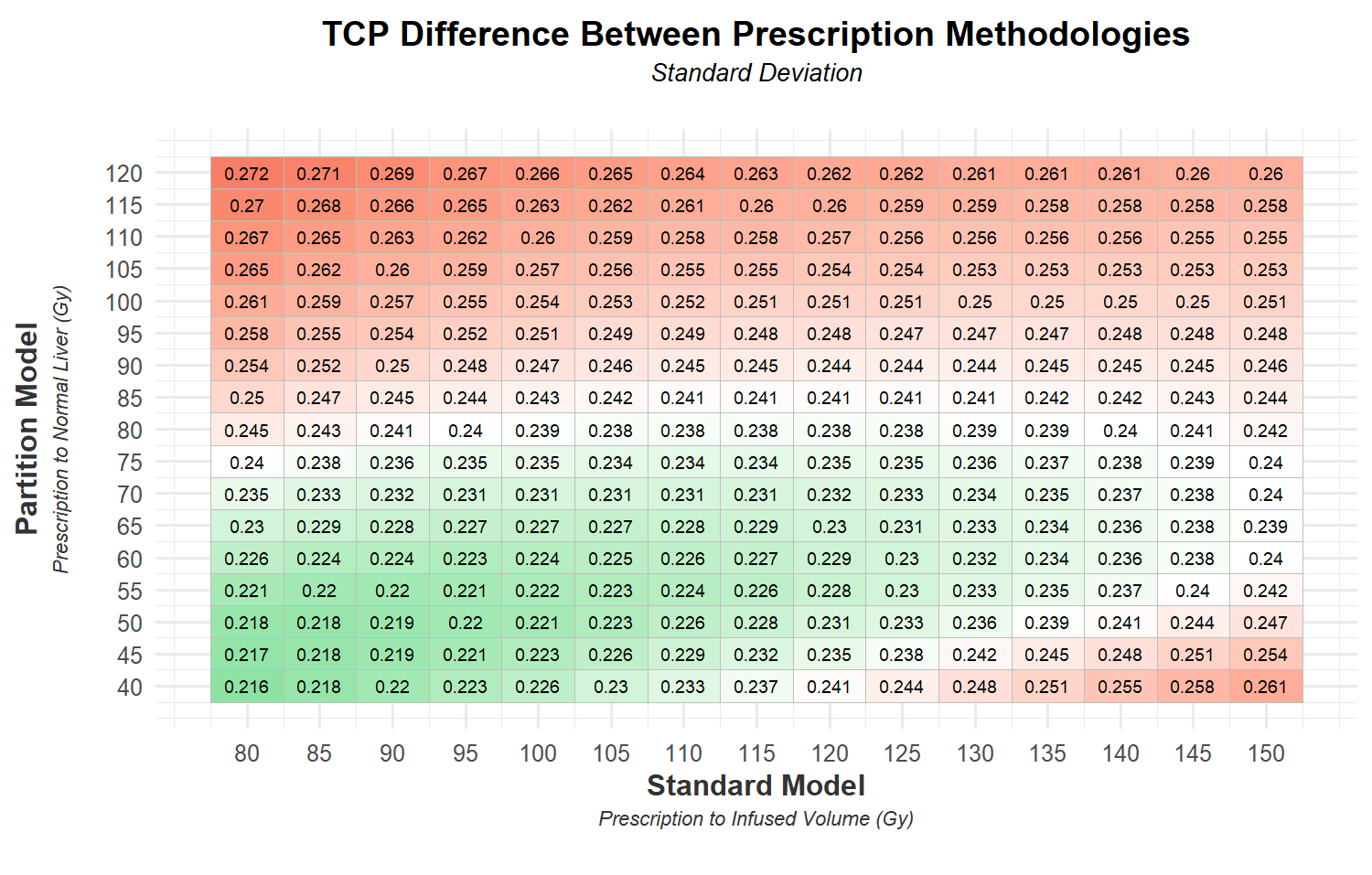


**Fig. S1** The standard deviation is shown for each average change in TCP calculated presented in figure 4.


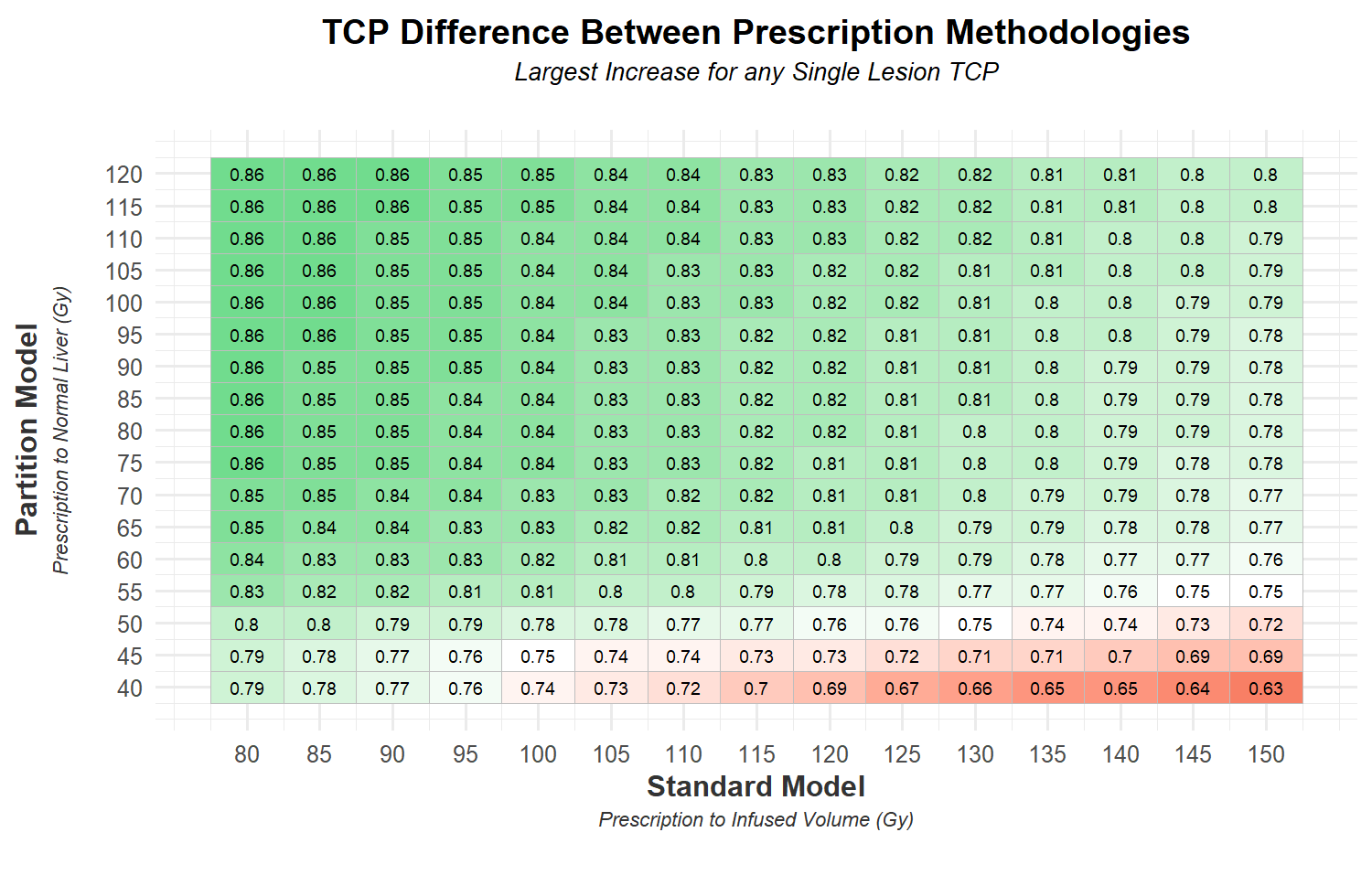


**Fig. S2** The largest increase in TCP for any lesion when changing from a standard model prescription to a partition model prescription.


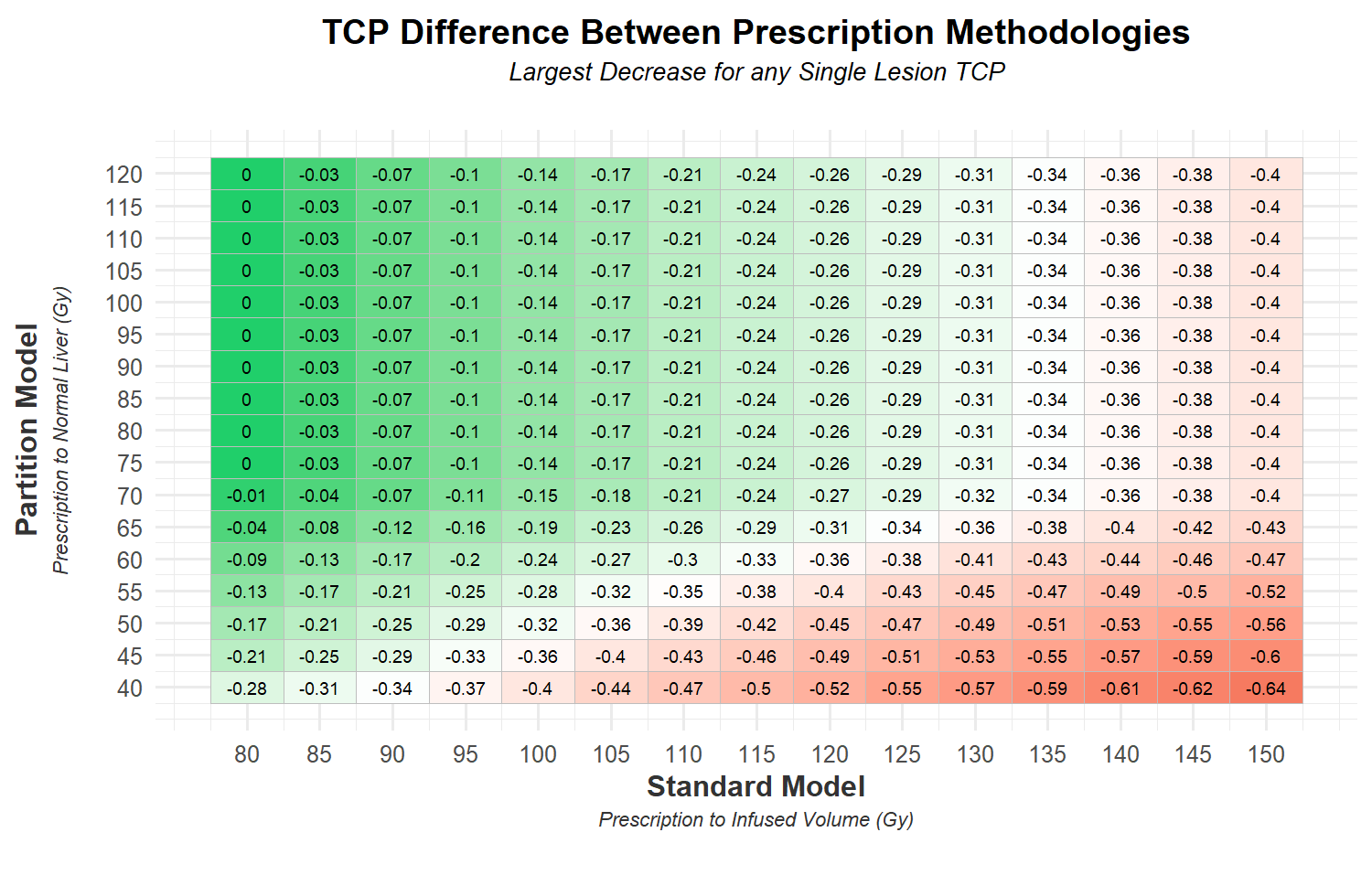


**Fig. S3** The largest decrease in TCP for any lesion when changing from a standard model prescription to a partition model prescription.
